# Challenging Cosmetic Innovation: The Skin Microbiota and Probiotics Protect the Skin from UV-Induced Damage

**DOI:** 10.3390/microorganisms9050936

**Published:** 2021-04-27

**Authors:** Djouhar Souak, Magalie Barreau, Aurélie Courtois, Valérie André, Cécile Duclairoir Poc, Marc G. J. Feuilloley, Manon Gault

**Affiliations:** 1 BASF Beauty Care Solutions France SAS, 69007 Lyon, France; djouhar.souak@basf.com (D.S.); aurelie.courtois@basf.com (A.C.); valerie.andre-frei@basf.com (V.A.); 2 LMSM EA4312, Laboratoire de Microbiologie Signaux et Microenvironnement, Université de Rouen Normandie, 27000 Evreux, France; magalie.barreau@univ-rouen.fr

**Keywords:** cosmetics, skin microbiota, next-generation sequencing, metabolites, probiotics, photoprotection

## Abstract

Many studies performed in the last decade have focused on the cutaneous microbiota. It has been shown that this microbiota plays a key role in skin homeostasis. Considered as “a second barrier” to the environment, it is very important to know how it reacts to exogenous aggressions. The cosmetics industry has a started to use this microbiota as a source of natural ingredients, particularly ones that confer photoprotection against ultraviolet (UV) rays. Interestingly, it has been demonstrated that bacterial molecules can block UV rays or reverse their harmful effects. Oral probiotics containing living microorganisms have also shown promising results in restoring skin homeostasis and reversing the negative effects of UV rays. Microbial-based active sunscreen compounds have huge potential for use as next-generation photoprotection products.

## 1. Introduction

The fields of cosmetics and dermatology have recently focused their studies on the cutaneous microbiota and its interaction with the skin and its environment. 

UV rays are one of the most concerning environmental factors affecting the skin. Many studies have focused on the effects of UV rays on the skin, but few have investigated the cutaneous microbiota. Some effects may be positive, such as vitamin D production, but most appear to be negative. Photoprotection is thus necessary. A recent claim from the cosmetics industry is that it is possible to improve physical or chemical sunscreens with substances showing natural UV-absorbing capacity and/or with molecules that mitigate the harmful effects of UV. The objective of this review is to consider and discuss the ability of cutaneous microorganisms to protect against damage caused by UV rays and their possible skin benefits. Formulations of cosmetic products containing probiotic and postbiotic ingredients are intended to block the effects of UV radiation effects and protect or restore the balance of the cutaneous microbiota due to their anti-oxidant and/or anti-inflammatory activities [1]. The term probiotic refers to live microorganisms, which, when administered in adequate amounts, confer a health benefit to the host [2]. Meanwhile, the term postbiotic refers to metabolic byproducts, such as enzymes, peptides, teichoic acid, peptidoglycan derived muropeptides, exopolysaccharides, cell surface and secreted proteins, bacteriocins and organic aids generated by a probiotic organism during its lifespan [3]. In this study we propose to identify protection strategies related to the cutaneous microbiota such as probiotics or postbiotics.

## 2. The Human Skin Microbiota

Emergence of “omic” technologies (including next-generation sequencing and highthroughput and sensitivity mass spectrometry) have provided a better global understanding of the skin microbiota. In the past, knowledge of the skin microbiota was limited to culture-dependent assays, although it is estimated that less than 1% of microbial species can be cultivated [4].

The skin is a complex and stratified organ providing very diverse ecological conditions (wet, dry or sebaceous, with a temperature between 33 and 37 °C and local oxygen concentrations ranging from 3 to 20%) [5]. This ecosystem (1.8 m^2^), one of the largest of the human body, harbors an equivalent diversity of inhabitants including bacteria, fungi, yeasts, archaea, viruses, and even mites [6,7]. From birth, an individual’s skin microbiota is formed by transfer from the vaginal flora after delivery, or by the environmental flora in the case of delivery by caesarean section [8]. This cutaneous microbiota will then evolve progressively, varying from one individual to another according to age, sex, genetic factors, physico-chemical factors (e.g., humidity, pH, temperature), the composition of antimicrobial peptides and lipids, the environment, lifestyle (cosmetic use), and immune status [6,8]. It will develop differently according to its location (e.g., face, armpits, back) and microenvironment [9].

**Figure 1:**
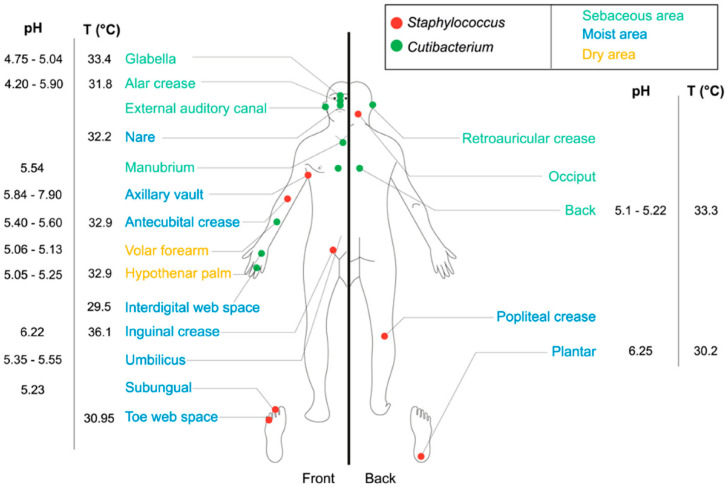
Topographical distribution of *Staphylococcus* and *Cutibacterium* on the human skin [22].

This microbiota is usually divided into commensal and transient bacteria. Transient microorganisms include opportunistic pathogens, whereas commensals are mostly considered to be beneficial, although they can also acquire a significant virulence under the effect of endogenous or exogenous factors [10]. The skin commensal microbiota has several key defense functions against pathogens, and functions as a barrier for (bio)chemical and physical aggression and a modulator of the skin innate (through antimicrobial peptides synthesis) and adaptive immune systems [7,11,12]. In fact, its composition is crucial for immune homeostasis [13]. A disruption of this balance can lead to diseases such as atopic dermatitis, psoriasis, rosacea or immediate and delayed hypersensitivity [9,14,15]. For instance, an increase in *Staphylococcus aureus* is correlated with the occurrence of atopic dermatitis [16,17,18], but the situation is not so simple, as atopic dermatitis is also associated with a large decrease in microbial diversity [19], highlighting the multifactor origin of most cutaneous diseases. Bacteria are the main components of the skin microbiota [20]. The composition and distribution of the bacterial microbiota on adult skin is regulated by the local parameters of the skin, and the population differs remarkably between wet, dry and sebaceous cutaneous regions. Considering samples collected over 20 skin sites, the major bacterial phyla appear to be Actinobacteria (51.8%), Firmicutes (24.4%), Proteobacteria (16.5%) and Bacteroidetes (6.3%), while all other phyla account for less than 1% [9]. The major identified genera are Corynebacteria (22.8%, Actinobacteria), Cutibacteria (former Propionibacteria) (23.0%, Actinobacteria) and Staphylococci (16.8%, Firmicutes). This bacterial community profile is an average, but rare bacterial phyla such as Cyanobacteria (2.5%) can be also identified and result probably from the interaction with the environment [21,22,23]. The abundance of each group is strongly dependent on the characteristics of the appropriate niche. For example, lipophilic species, such as *Cutibacterium acnes*, are found in sebaceous sites, whereas *Staphylococci* develops mostly in humid areas [4,19,24] (Figure 1).

The composition of the bacterial microbiota is also modulated by the skin microenvironment, including factors such as oxygen, pH, temperature and topography (see Figure 1) [7,22]. For instance, following the local oxygen partial pressure, anaerobic bacteria can be found deep in the hair follicle, while aerobic bacteria are localized in external layers of the *Stratum corneum* [7]. The epidermal surface is regularly renewed and is not a favorable environment for microorganisms, with each squama taking with it a mean of 30 bacteria [25]. Cutaneous bacteria tend to develop beneath the surface and particularly in cutaneous annexes, such as the hair follicles, sweat glands, and sebaceous glands. As a result, 25% of the total bacterial microflora grow under the skin surface [26,27].

Concerning yeasts and fungi, studies are more limited, but have identified *Malassezia* as the predominant microorganism on the body and arms. Other microorganisms, including *Aspergillus*, *Cryptococcus*, *Rhodotorula* and *Epicoccum* can be found on the arch of the foot [24,28]. A total of 17 species of *Malassezia* have been identified on the human skin, but *M. restricta*, *M. globosa* and *M. sympodialis* are the principal ones [28].

Archaea were ignored for a long time as members of the human cutaneous microbiota, but studies concerning them appeared in 2013 [29], revealing that they could represent 4.2% of the microbiota in certain areas such as the torso. Identified species include soil group archaea, methanogens and halophils [30]. Currently, their function in the skin remains unknown.

The average population of viruses on the skin is estimated to be 10^6^/cm^2^ [31]. The cutaneous virome can be separated into two groups. The first group is composed of bacteriophages, whose distribution follows that of their host’s bacteria [32]. The second group is composed of eukaryote viruses, including *Polyoma*, *Papilloma* and *Circoviruses*, which can be found even in the absence of clinical signs of infection [33,34].

Interestingly, this cutaneous microbiota is exposed every day to stressful environmental factors, such as unfavorable temperatures, humidity, the sun, air pollution, textile leachables, drugs, disinfectants and even cosmetics, which can all cause dysbiosis (changes in the microbiota balance) and consequently skin disorders [19].

## 3. Impact of UVs on the Skin and Its Microbiota

### 3.1. Effect of UVs on the Skin

Sun exposure can affect a variable percentage of the skin surface depending on the seasons, but it remains one of the most powerful and constant sources of environmental stress for the skin. The solar radiation spectrum covers wavelength starting from ultraviolets (UV; 100–400 nm) to the infrared region (>740 nm), including the visible band (400–740 nm). The energy transmitted by solar radiation is inversely correlated to the wavelength. Simply put, long wave radiation contains less energy than short wave radiation, such as UV. UV radiation represents the highest energy component of the electromagnetic spectrum that reaches the terrestrial atmosphere. The solar UV spectrum is itself divided into three segments based on the wavelength and energy transmitted by the radiation: UVC (200–290 nm); UVB (280–315 nm) and UVA (315–400 nm), with the latter being divided into UVA1 (315–340 nm) and UVA2 (340–400 nm) [35,36,37,38].

**Figure 2:**
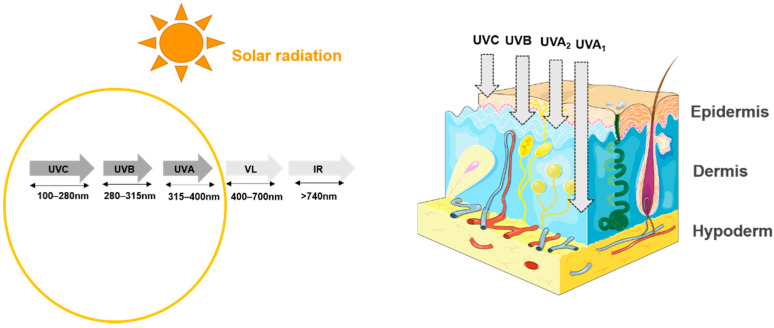
Spectrum of solar radiation. VL: visible light; IR: infrared-ultraviolet radiation accounts for 58% of the total solar spectrum and is divided into three groups, from the shortest to longest wavelength: UVC (100–280 nm), UVB (280–315 nm), and UVA (315–400 nm). Each group penetrates the skin to different depths [38].

The effects of UV radiation on the skin differ depending on their energy and potential for penetration (Figure 2). The longer the radiation wavelength is, the deeper it will penetrate into the skin. UVC is efficiently absorbed by ozone in the stratosphere. At sea level, humans are mainly exposed to UVB and UVA. According to the literature, UVs that penetrate the skin represent 5 to 8% of sunlight radiation corresponding to approximately 5–10% UVB and 90–95% UVA [38,39,40,41]. These values vary with the sun elevation, altitude, ozone, cloud covering and ground reflection. Pollution and stratospheric ozone layer depletion can, however, magnify ultraviolet radiation exposure values [37].

The beneficial or harmful effects of the sun on the skin, and especially the impact of solar UV radiation, have been widely studied [41,42]. Six skin phototypes have been described by Fitzpatrick according to their level of skin pigmentation and sensitivity to UV radiation [43]. In the skin, UV radiation is absorbed by cutaneous chromophores as well as different molecules, including DNA, membrane lipids, and trans-urocanic acid [44]. This diversity of targets explains the large panel of known biological responses [44]. In addition to psychological effects, sunlight and UV advantageously promote vitamin D synthesis and their beneficial influence on well-being was at the origin of the practice of phototherapy. Phototherapy is prescribed in skin pathologies, such as psoriasis, atopic dermatitis, cutaneous T-cell lymphoma and other photoresponsive dermatoses [36].

However, excessive or long-term exposure to UV radiation can also have harmful effects. Solar exposure generates a complex skin response, including histological changes leading to the premature aging of the skin and even carcinogenesis [41,45]. In fact, UV radiation has different targets and activities. UVA targets DNA and produces free radicals, which promote lipid oxidation and lead to inflammation and long-term gene expression and immune response changes. By contrast, UVB is essentially absorbed by nuclear DNA and causes immediate damage [41].

### 3.2. Effect of UVs on the Cutaneous Microbiota

Few studies have focused on the effects of UVs on the cutaneous microbiota. However, the effects of UVs on the skin can indirectly affect the cutaneous microbiota and bacteria themselves have developed resistance to UV radiation.

It has been demonstrated that UV radiation influences the composition and activity of the cutaneous microbiota, but its consequences are ambiguous, ranging from positive, such as causing a decrease in opportunistic pathogens such as *Staphylococcus aureus* [36], to negative, with the appearance of chronic inflammation [45].

UV radiations can upregulate skin protective innate immune mechanisms by stimulating the production of antimicrobial peptides, such as hbD2, hbD3, RNase7, psoriasin or S100A12, by keratinocytes [11].

Recently, the impact of UVA and UVB on the cutaneous microbiota was studied in a panel of six volunteers. An alteration in the composition of their cutaneous microbiota was observed after exposure to UVA and UVB. Although the changes were highly variable, the phylum *Cyanobacteria* tended to increase while *Lactobacillaceae* and *Pseudomonadaceae* decreased. The increase in *Cyanobacteria* was attributed to their high intrinsic resistance to UV radiation [20]. Indeed, *Cyanobacteria* develop a diversity of defense mechanisms including the biosynthesis of UV-absorbing/screening compounds, such as mycosporine-like amino acids (MAAs), and enzymes, including superoxide dismutases (SOD), which counteract oxidative stress [23]. UV rays also directly affect cutaneous bacteria such as *Cutibacterium acnes* by reducing their porphyrin production [46]. UV also acts on another common skin bacterium, *Micrococcus luteus*. This strain has the remarkable property of being able to antagonize the deleterious effect of UV on the immune system through the reversion of cis-urocanic acid formed by UVs during skin exposure [47].

This has led the cosmetic industry to take into consideration the cutaneous microbiota in the development of products used for photoprotection.

## 4. Photoprotection

### 4.1. Classical Sun Protection

The skin has natural photoprotection against UV rays owing to its pigmentation, antioxidant enzymes, and DNA repair system with base and nucleotide excision repair (BER, NER) and even apoptosis [48]. However, particularly during the hours of maximal exposure to the sun between 10 a.m. and 2 p.m., photoprotection is necessary in order to protect against the deleterious effects of UV radiation on the skin. The first strategy to achieve protection is wearing clothes made of cloth that provide UV protection. UV rays can penetrate the thin cotton clothes generally worn in the summer [37]. Modern photoprotection includes two factors as means of primary and secondary protection. At the primary level, we find sunscreens (regulated at the FDA level) that absorb or reflect UV radiation. At the secondary level, we find antioxidants, osmolytes and DNA repair enzymes, which help in limiting skin damage [49,50].

Sunscreens are the most popular means of cutaneous protection against UVs in Western countries [51]. Chemical sunscreens are known to compensate for the weakness of natural photoprotection, particularly for Caucasian skin (phototypes I to IV) [52]. Some physically limit the penetration of UV radiation into the cellular target by blocking radiation (classical sun products, UV filters). Other cosmetic products additionally target the effects of UVs by inhibiting UV induced oxygen-reacting species or have more complex actions, such as promoting endogenous photoprotection and reparation systems [51,53].

More precisely, sunscreens can be divided based on their mechanism of action into physical UVs blockers (inorganic), chemical UVs absorbers and hybrid UV filters [1,49,54].

Inorganic (physical) UV filters reflect and scatter light and particularly UVA and UVB. These filters include color compounds and micronized pigments. Amongst the latter, we find titanium oxide (TiO_2_) and zinc oxide (ZnO ex Z-Cote^®^-BASF Care Creations). Organic (chemical) UV filters (such as Tinosorb^®^ M-BASF Care Creations, Mexoryl^TM^ XL-L’Oréal, Triasorb™-Pierre Fabre) absorb high-energy UV rays and release lower energy radiation thanks to a chromophore that is typically an aromatic moiety conjugated or not to a carbonyl group [23,49,54]. Neither of these filters penetrate the skin barrier nor enter the cells, where they could cause mutations, nor do they reach the systemic circulation [37].

However, protecting the skin only against UV rays is not sufficient, because recent studies have shown that synergistic effects can exist between UV and atmospheric pollutants such as cigarette smoke and polycyclic aromatic hydrocarbons (PAHs) [55]. These pollutants have intrinsic skin oxidative properties whose effects can potentiate those of UV rays. This was particularly demonstrated for UVA and B[a]P (Benzo[a]pyrene), one of the most harmful photo-reactive PAHs, leading to a decrease in cell viability through an increase in lipid peroxidation and DNA breakages [55]. Thus, the combination of sunlight and air pollutants has been proven to synergistically aggravate skin damage and accelerate skin aging [55].

Complementary to UV filters, strategies based on endogenous protection against environmental synergistic damage have been implemented by stimulating natural antioxidant pathways or adding, for example, repairing enzymes, antioxidants, peptides [35,36,56], or natural or biotechnological extracts [1,37,53] (Table 1). Antioxidants, such as vitamin C, vitamin E, carotenoids, polyphenols and flavonoids allow a reduction of UV-generated reactive oxygen species [1]. This is the case for Ciste’M^®^ (BASF Beauty Care Solutions France), a natural extract of the aerial parts of *Cistus monspeliensis* titrated in flavonoids myricetin glycosides. In vitro studies have demonstrated the broad biological protective activity of Ciste’M^®^ against the deleterious effects of UVB radiation, UVA radiation and benzo[a]pyrene through associated anti-inflammatory, antioxidants and DNA protective properties. Another example is Arganyl^®^ (BASF Beauty Solutions Care France), whose active ingredient is derived from argan leaves, known for their high concentrations of polyphenols. It exhibits an anti-oxidative effect by inhibiting the production of reactive oxygen species and lipid peroxidation induced by benzo[a]pyrene and UVA.

### 4.2. Bacterial-Based Cosmetic Approaches to Preventing UV-Induced Damages

As previously mentioned, mycosporine-like amino acids (MAAs) are natural photostable secondary class metabolites and UV-absorbing compounds produced by lichens, fungi and *Cyanobacteria* upon exposure to solar UV rays. Commonly termed “microbial sunscreens”, MAAs can dissipate UV energy as heat without generating free oxygen radicals and can also block the UV-induced formation of both (6–4) pyrimidine-pyrimidone photoproducts (6–4 photoproducts) and pyrimidine dimers [54,57,58,59]. These UVs photoproducts lead to the induction of mutations, cell transformations and cell death [60]. MAAs are widely considered to be multifunctional compounds offering protection against UV radiation-induced damage as well as oxidative, osmotic and thermal stress. These molecules absorb light over a wide bandwidth with a maximum absorbance of between 310 and 362 nm (UVA and UVB range) and have a high molar extinction coefficient (e = 28,100–50,000 M^−1^ cm^−1^). Thus, MAAs may be used as active ingredients in cosmetic products to counteract the negative effects of solar UV radiation [58,61,62].

MAAs are typically small compounds (<400 Da), colorless and water-soluble. Twenty forms of MMAs have been identified and the most studied ones are porphyra-334, shinorine and mycosporin glycine. These compounds have a similar structure, composed of a 4-deoxygadusol containing cyclohexanone or cyclohexenimine rings conjugated to the nitrogen substituent of an amino acid or imino alcohol [23]. Many studies have shown that MAAs are antioxidants, as they specifically counteract oxidative damages by preventing lipid peroxidation and superoxide radical activity. For example, red algae *Porphyra umbilicalis* extract, commercialized as Helioguard 365a (Mibelle AG Biochemistry, Switzerland), is claimed to be a natural sunscreen and contains a blend of liposomal MAAs, shinorine and porphyra-334 [61,63] (Table 1). The phoprotective action of this compound against DNA damage caused by UVA has been demonstrated in vitro in HaCaT cells [61].

Recent studies highlight a new function of skin bacteria as protectors against external aggression, such as UV radiation. It has been demonstrated that *Staphylococcus epidermidis* produces a compound, 6-HAP (6-N hydroxyaminopurine), which has a protective activity against neoplasia. This molecule is able to inhibit DNA synthesis and to selectively prevent the proliferation of tumor cells, as well as to suppress UV induced de novo cell growth [63].

UVB radiation is known to depress cell-mediated immunity through the photoisomerization of *trans*-urocanic acid (*trans*-UCA) to *cis*-urocanic acid (*cis*-UCA). It has also been demonstrated that the skin commensal *Micrococcus luteus* is able to degrade *cis*-UCA into its trans isoform and thus potentially reduce the immunosuppressive action of UVB [45,47]. Therefore, the deleterious effects of UV radiation may be mitigated by this bacterial species. In addition, *Micrococcus luteus* produces in particular an interesting enzyme, an endonuclease, which has the capacity to improve the efficiency of DNA repair enzymatic complexes. This endonuclease can be encapsulated in a phospholipid-coated envelope to facilitate its penetration into cells [37].

To limit DNA damage, another type of enzymes, photolyases, are of major interest in the field of photoprotection. These enzymes are produced by many animal species, plants and bacteria naturally exposed to UV radiation but are not encoded in placental mammals, including humans [64]. Photolyases belong to a class of 50–60 kDa flavoproteins activated by blue or violet light of the visible spectrum. They are able to counteract the formation of UV-generated DNA photoproducts, such as cyclobutane pyrimidine dimers (CDPs) and 6–4 photoproducts, as previously mentioned (Section 3.1). Photolyases are specific to one type of product, but their mechanism of action remains unclear. However, they have a remarkable activity [47,65]. Authors have shown that a topical treatment of human skin with liposomes containing a photolyase isolated from a cyanobacterium, *Anacystis nidulans,* was capable of degrading 40% of the CDPs generated by UVB exposure, as well as reducing erythema. In parallel, a reduction of intercellular adhesion molecule-1 (ICAM-1), acting on immunity and inflammation was noted in the epidermis. Moreover, photolyases appear effective in reducing the deleterious effects of UVB and generating immunoprotection [65,66].

A recent discovery showed that the skin commensal bacterium, *Cutibacterium acnes*, was able to secrete an antioxidant enzyme [67]. This protein named RoxP for the Radical oxygenase of *Propionibacterium acnes* facilitates aerobic bacterial growth in vitro and ex vivo. Another study has shown that RoxP positively influences the viability of monocytes and keratinocytes exposed to oxidative stress [68]. This enzyme has interesting properties and could be of interest in reducing oxidative stress linked to UV exposure.

*Actinobacteria* and, more particularly, *Streptomyces* are sources of metabolites with very interesting activities in photoprotection, such as antioxidant and anti-inflammatory compounds and UV-absorbing molecules [69]. These molecules include amide compounds, generally associated with anti-inflammatory activities and alkaloids showing more specifically antioxidant activities. These compounds are now employed as chemical ingredients to develop protective products [69].

### 4.3. Bacterial-Based Nutritional Approaches to Prevent UV-Induced Damages

Varieties of dietary supplements are now known for their benefits for skin health [70]. Oral supplementation with antioxidants (e.g., ascorbic acid, carotenoids or polyphenols) and probiotics was recently proposed to protect skin against UV radiation-induced damage [70,71].

The term “probiotic” was defined by Ruler in 1989 as “living microorganisms, which, when consumed in adequate amounts, confer a health effect on the host” [72,73]. Specific strains of lactic acid bacteria may have a beneficial influence on the composition and metabolism of the intestinal microbiota and in some cases have been reported to inhibit the growth of enteropathogenic bacteria [72]. Many studies have shown a connection between the intestinal immune axis and the skin [74] and consumption of food containing probiotics was shown to improve skin health, maintaining skin homeostasis and regulating the cutaneous immune system [49].

With regard to UV radiation-induced skin damage, the efficiency of probiotics such as *Lactobacillus johnsonii* NCC 533 (La1) has been demonstrated. The absorption of *L. johnsonii* was shown to reinforce cutaneous immune system homeostasis by preventing the increase in interleurkin-10 generated by UVs and to decrease the recruitment of epidermal Langerhans cells [75,76]. In the same way, the administration of a probiotic strain of *Lactobacillus rhamnosus* GG (LGG) was demonstrated to prevent the development of skin tumors thanks to the activity of its lipoteichoic acid (LTA), a component of the Gram-positive bacteria cell wall. In a murine model, LTA decreased UV-induced skin immunosuppression and thus significantly reduced UV-induced skin tumor growth [74]. Other photoprotective candidates can be considered, such as *Lactobacillus plantarum* HY7714, *Bifidobacterium breve* and *Bifidobacterium longum* [75,77,78]. These observations are promising but need to be confirmed in humans.

## 5. Conclusions

There are several photoprotection strategies currently on the market. Avoiding exposure to sunlight and protecting the skin using clothing as well as sunscreen are currently the best strategies. However, optimized strategies could either use ingredients improving the endogenous protective response to UV rays and/or repair or antioxidant enzymes with a positive effect on the recovery of the skin after UV exposure [1,79].

As described above, the cutaneous microbiota is considered as a source of compounds with indirect photoprotective properties. Some bacteria of the cutaneous microbiota even have direct UV radiation blocking or absorbing effects, as well as anti-inflammatory and anti-oxidative activities.

In addition, some clinical studies have reinforced the idea that certain probiotics have beneficial activities that can prevent or reverse the harmful effects of UV radiation [49]. Endogenous and exogenous bacteria are not only a source of molecules but also a source of inspiration for the development of new natural photoprotection strategies.

## Figures and Tables

**Table 1:** Non-exhaustive list of marketed cosmetic ingredients related to photoprotection according to their mechanisms of action. I.N.C.I.: International Nomenclature of Cosmetic Ingredients.

Cosmetic Ingredient	Nom I.N.C.I.	Mechanism of Action
Inoveol^®^ CAFA (Sederma)	Thermus Thermophilus Ferment (and) Glycerin	Protection against UV and Infrared (IR). Anti-aging protection.
Sun’Alg^®^ (Biosil Technologies Inc.)	Pongamia glabra seed oil (and) Dunaliella salina extract (and) Haematococcus pluvialis extract	Shield formation against UVA and UVB radiation (natural sunscreen thanks to its absorption). Two microalgal extracts: additional protective capacity against the oxidative stress (mixed carotenoids composition).
Arganyl^®^ (BASF Beauty Care Solutions)	Argania Spinosa Leaf Extract (and) Maltodextrin	Anti-oxidative efficacy by inhibiting Reactive oxygen species production lipid peroxidation induced by pollutants (PM2.5, B[a]P) and UVA.
Chronogen^TM^ (Ashland)	Water (aqua) (and) glycerin (and) hydrolyzed yeast protein	Repair of UV damage. Decrease in induced sunburned cells.
Ciste’M^®^ (BASF Beauty Care Solutions)	Maltodextrin (and) Cistus Monspeliensis Flower/Leaf/Stem Extract	Protection against the deleterious effects of UVB, UVA radiation and benzo[a]pyrene. Anti-inflammatory. Antioxidants. DNA protective properties.
Helioguard^TM^365 (Mibelle AG Biochemistry)	Aqua/Water (and) Lecithin (and) Alcohol (and) Sodium Lactate (and) Porphyra Umbilicalis Extract (and) Phenoxyethanol	UVA bioprotector (UVA induced skin alterations). Prevents premature photoaging. Limits damage to DNA and lipids.
